# Staphylococcus aureus Coproporphyrinogen III Oxidase Is Required for Aerobic and Anaerobic Heme Synthesis

**DOI:** 10.1128/mSphere.00235-19

**Published:** 2019-07-10

**Authors:** Jacob E. Choby, Eric P. Skaar

**Affiliations:** a Department of Pathology, Microbiology, and Immunology, Vanderbilt University Medical Center, Nashville, Tennessee, USA; b Graduate Program in Microbiology and Immunology, Vanderbilt University, Nashville, Tennessee, USA; c Vanderbilt Institute for Infection, Immunology, and Inflammation, Vanderbilt University Medical Center, Nashville, Tennessee, USA; University of Nebraska Medical Center

**Keywords:** *Staphylococcus*, heme, tetrapyrroles

## Abstract

The virulence of the human pathogen Staphylococcus aureus is supported by many heme-dependent proteins, including key enzymes of cellular respiration. Therefore, synthesis of heme is a critical component of staphylococcal physiology. S. aureus generates heme via the coproporphyrin-dependent pathway, conserved across members of the *Firmicutes* and *Actinobacteria*. In this work, we genetically investigate the oxidation of coproporphyrinogen to coproporphyrin in this heme synthesis pathway. The coproporphyrinogen III oxidase CgoX has previously been identified as the oxygen-dependent enzyme responsible for this conversion under aerobic conditions. However, because S. aureus uses heme during anaerobic nitrate respiration, we hypothesized that coproporphyrin production is able to proceed in the absence of oxygen. Therefore, we tested the contribution to anaerobic heme synthesis of CgoX and two other proteins previously identified as potential oxygen-independent coproporphyrinogen dehydrogenases, NWMN_1486 and NWMN_1636. We have found that CgoX alone is responsible for aerobic and anaerobic coproporphyrin synthesis from coproporphyrinogen and is required for aerobic and anaerobic heme-dependent growth. This work provides an explanation for how S. aureus heme synthesis proceeds under both aerobic and anaerobic conditions.

**IMPORTANCE** Heme is a critical molecule required for aerobic and anaerobic respiration by organisms across kingdoms. The human pathogen Staphylococcus aureus has served as a model organism for the study of heme synthesis and heme-dependent physiology and, like many species of the phyla *Firmicutes* and *Actinobacteria*, generates heme through a coproporphyrin intermediate. A critical step in terminal heme synthesis is the production of coproporphyrin by the CgoX enzyme, which was presumed to be oxygen dependent. However, S. aureus also requires heme during anaerobic growth; therefore, the synthesis of coproporphyrin by an oxygen-independent mechanism is required. Here, we identify CgoX as the enzyme performing the oxygen-dependent and -independent synthesis of coproporphyrin from coproporphyrinogen, resolving a key outstanding question in the coproporphyrin-dependent heme synthesis pathway.

## Abstract (precis)

Heme is a critical molecule required for aerobic and anaerobic respiration by organisms across kingdoms. The human pathogen Staphylococcus aureus has served as a model organism for the study of heme synthesis and heme-dependent physiology and, like many species of the phyla *Firmicutes* and *Actinobacteria*, generates heme through a coproporphyrin intermediate. A critical step in terminal heme synthesis is the production of coproporphyrin by the CgoX enzyme, which was presumed to be oxygen dependent. However, S. aureus also requires heme during anaerobic growth; therefore, the synthesis of coproporphyrin by an oxygen-independent mechanism is required. Here, we identify CgoX as the enzyme performing the oxygen-dependent and -independent synthesis of coproporphyrin from coproporphyrinogen, resolving a key outstanding question in the coproporphyrin-dependent heme synthesis pathway.

## OBSERVATION

Staphylococcus aureus is an important human pathogen responsible for a wide variety of diseases in numerous host niches (1) and is supported by a robust host-adapted metabolism. S. aureus is a facultative anaerobe of the *Firmicutes* phylum capable of performing oxygen-dependent cellular respiration or transitioning to anaerobic respiration in the presence of alternative terminal electron acceptors like nitrate. In the absence of respiration, fermentation of a variety of carbon sources supports S. aureus replication. The tetrapyrrole cofactor heme is important for the cellular processes of many organisms across kingdoms, and in S. aureus, heme is required for aerobic and anaerobic respiration. Heme is a cofactor of the succinate dehydrogenase (SdhC; cytochrome *b_558_* subunit [2, 3]) that reduces menaquinone, the two terminal cytochrome oxidases (QoxABCD and CydAB [4, 5]) that oxidize menaquinol during aerobic respiration, and the nitrate reductase (NarI; gamma subunit [6, 7]) that oxidizes menaquinol during anaerobic nitrate respiration. Therefore, mutants unable to synthesize heme are respiration deficient and grow poorly, adopting a “small-colony variant” phenotype most evident on solid medium (8). Additionally, S. aureus and other species rely on heme for the function of many widely conserved enzymes, including catalase, nitric oxide synthase, and globin family proteins.

Bacteria synthesize heme from the universal precursor δ-aminolevulinic acid (ALA) which proceeds to uroporphyrinogen. Uroporphyrinogen can be diverted to form similar cofactors, including siroheme for use in the S. aureus nitrite reductase (9). Bacteria use different pathways to synthesize heme from uroporphyrinogen, which has been extensively reviewed by Dailey and colleagues (10). In only the last decade, it has been recognized that species of *Firmicutes* and *Actinobacteria* synthesize heme via a unique pathway that uses the coproporphyrin intermediate (11, 12). The identification of coproheme decarboxylase as a heme synthesis enzyme was a major step in understanding this newly appreciated pathway (13–15). However, the enzyme responsible for coproporphyrin production from coproporphyrinogen in the absence of oxygen has not been defined.

## 

### The anaerobic conversion of coproporphyrinogen to coproporphryin is an outstanding question in coproporphyrin-dependent heme synthesis.

**FIG 1:**
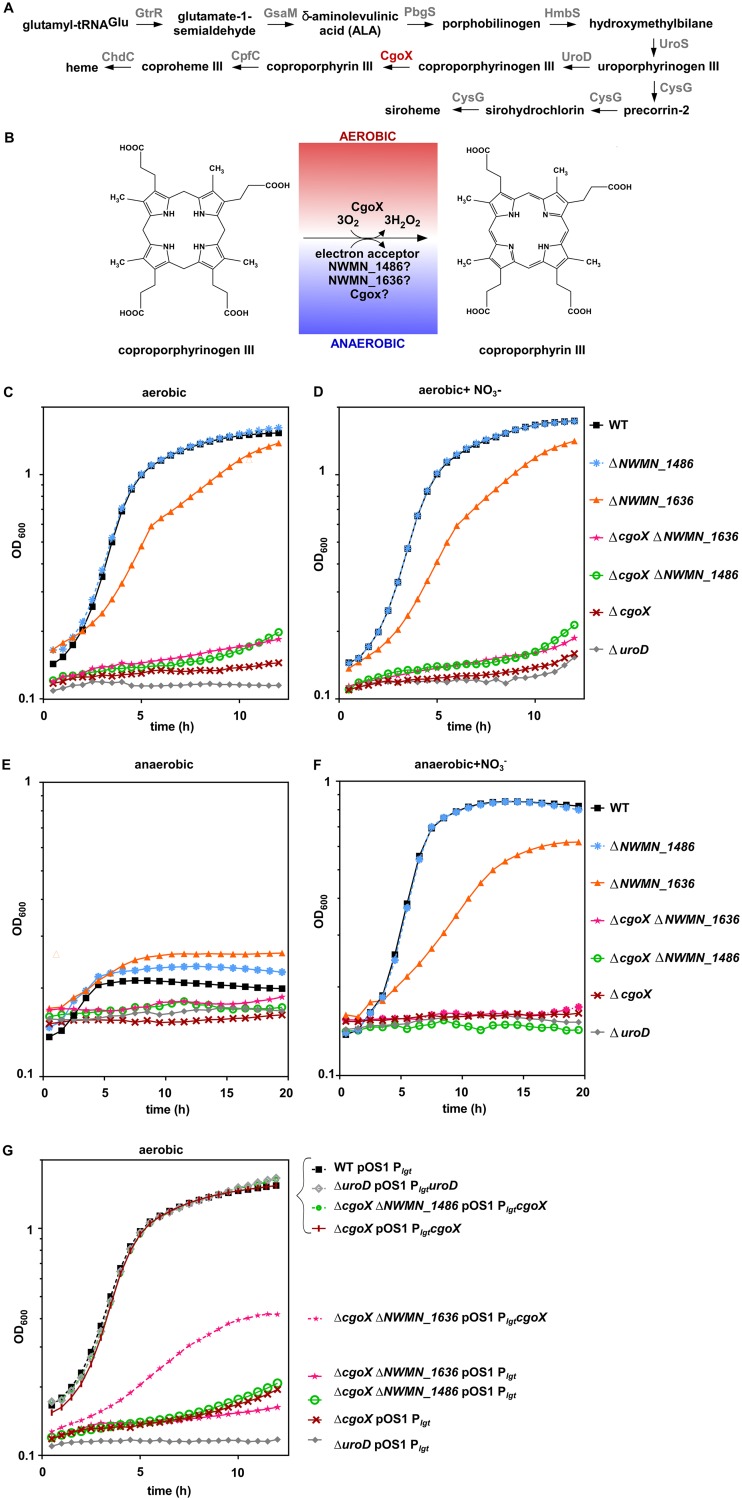
CgoX is required for aerobic and anaerobic heme synthesis. (A) Overview of heme synthesis in S. aureus. (B) Details of the conversion of coproporphyrinogen to coproporphyrin performed aerobically by CgoX and potential enzymes functioning anaerobically. (C to F) Growth of S. aureus WT and indicated mutants in medium containing glycerol as the primary carbon source under the following conditions: aerobically (C), aerobically and supplemented with nitrate (D), anaerobically (E), and anaerobically and supplemented with nitrate (F). (G) Aerobic growth of WT and indicated mutants encoding plasmids with constitutive promoter or constitutive promoter upstream of *uroD* or *cgoX* in medium containing glycerol as the primary carbon source. OD_600_, optical density at 600 nm.

In S. aureus and other organisms that synthesize heme via coproporphyrin, UroD produces coproporphyrinogen (Fig. 1A) from the common precursor uroporphyrinogen. Coproporphyrinogen oxidase (CgoX) has been identified as the next enzyme in the pathway (Fig. 1A). CgoX performs the oxidation of coproporphyrinogen III to coproporphyrin (Fig. 1B), and *in vitro* this reaction uses molecular oxygen as the electron acceptor (12, 16–18). This suggests the existence of an enzyme capable of producing coproporphyrin anaerobically, using a different electron acceptor. Existence of separate oxygen-dependent and -independent enyzmes for this step in S. aureus would be in line with the oxygen-dependent and oxygen-independent synthesis of protoporphyrin IX in the protoporphyrin-dependent heme synthesis pathway (10). Based on literature and bioinformatic analyses, three different possibilities exist to resolve this gap in coporporphyin-dependent heme synthesis: HemN (NWMN_1486) acts as an oxygen-independent coproporphyrinogen III dehydrogenase, the DUF1444 protein (NWMN_1636) acts as an oxygen-independent coproporphyrinogen III dehydrogenase, or CgoX functions both anaerobically and aerobically (Fig. 1B). NWMN_1486 is annotated as an oxygen-independent coproporphyrinogen III oxidase in bioinformatic databases, making it an obvious candidate for this enzymatic step because it contains a radical *S*-adenosyl-l-methionine motif, a HemN fold, and high amino acid conservation to the prototypical HemN proteins that perform the parallel step in the protoporphyrin-dependent heme pathway (19, 20). Recent evidence from other species suggests that this annotation is likely incorrect; based on sequence homology, NWMN_1486 likely belongs to a protein family which functions as heme chaperones (11, 21, 22). However, the function of NWMN_1486 has not been studied. On the other hand, NWMN_1636 is a protein of unknown function but has been proposed as the possible oxygen-independent coproporphyrinogen III oxidase based on genomic context (10). This suggestion was included in the findings of a large-scale analysis of the coproporphyrin-dependent pathways across *Firmicutes* and *Actinobacteria*; a protein with the DUF1444 domain was found to cooccur with terminal heme synthesis enzymes in *Firmicutes* (10). Therefore, we set out to test these possibilities and determine which enzyme produced coproporphyrin anaerobically.

### CgoX is required for aerobic and anaerobic heme synthesis.

To experimentally test the contribution of CgoX, NWMN_1486, and NWMN_1636 to heme synthesis, in-frame unmarked deletions were created in each corresponding gene, as well as in *uroD.* We selected *uroD* as a control; a mutant of this gene is unable to synthesize heme but can still synthesize siroheme (Fig. 1A), the cofactor of nitrite reductase. S. aureus strains were grown in RPMI medium (without glucose), supplemented with Casamino Acids and glycerol as the primary carbon source, to quantitatively assess aerobic and anaerobic heme-dependent growth. As glycerol is nonfermentable, growth in this medium relies largely on oxygen-dependent (aerobic) or nitrate-dependent (anaerobic) respiration. S. aureus wild type (WT) grew robustly in this medium in the presence of oxygen, but the heme-deficient Δ*cgoX* and Δ*uroD* mutants were unable to grow (Fig. 1C; see also Fig. S1A in the supplemental material). The Δ*NWMN_1486* mutant grew robustly while the Δ*NWMN_1636* mutant had a modest growth defect. Under these conditions, addition of nitrate did not enhance growth as oxygen is the preferred terminal electron acceptor (Fig. 1D and Fig. S1B). Under anaerobic conditions, the Δ*uroD* mutant was unable to grow in the absence (Fig. 1E) or presence (Fig. 1F) of nitrate, consistent with heme being required for anaerobic growth under these conditions. Surprisingly the Δ*cgoX* mutant was also unable to grow when nitrate was provided, suggesting that CgoX is also required for anaerobic heme-dependent growth (Fig. 1E and F and Fig. S2). The Δ*NWMN_1486* and Δ*NWMN_1636* mutants were able to grow anaerobically when nitrate was provided, which is consistent with CgoX functioning aerobically and anaerobically, with no other gene being required for anaerobic conversion of coproporphyrinogen to coproporphyrin. As observed aerobically, the Δ*NWMN_1636* mutant had a modest growth defect anaerobically. Deletion of *cgoX* in the Δ*NWMN_1486* and Δ*NWMN_1636* mutant backgrounds reduced growth to the level of the Δ*cgoX* mutant alone. The growth of mutants lacking either *cgoX* or *uroD* was complemented by expressing the respective genes in *trans* (Fig. 1G and Fig. S3). Together, these data confirm that CgoX is required for anaerobic and aerobic growth under conditions which require heme and that NWMN_1486 and NWMN_1636 do not participate in anaerobic heme synthesis.

10.1128/mSphere.00235-19.1 FIG S1 Growth curves of Fig. 1C and D shown with standard errors of means. Download FIG S1, TIF file, 0.6 MB. Copyright © 2019 Choby and Skaar. 2019 Choby and Skaar This content is distributed under the terms of the Creative Commons Attribution 4.0 International license.

10.1128/mSphere.00235-19.2 FIG S2 Growth curves of Fig. 1E and F shown with standard errors of means Download FIG S2, TIF file, 0.7 MB. Copyright © 2019 Choby and Skaar. 2019 Choby and Skaar This content is distributed under the terms of the Creative Commons Attribution 4.0 International license.

10.1128/mSphere.00235-19.3 FIG S3 Growth curve of Fig. 1G shown with standard errors of the means. Download FIG S3, TIF file, 0.5 MB. Copyright © 2019 Choby and Skaar. 2019 Choby and Skaar This content is distributed under the terms of the Creative Commons Attribution 4.0 International license.

### Anaerobic production of heme relies only on CgoX, which is functionally conserved among *Firmicutes*.

**FIG 2:**
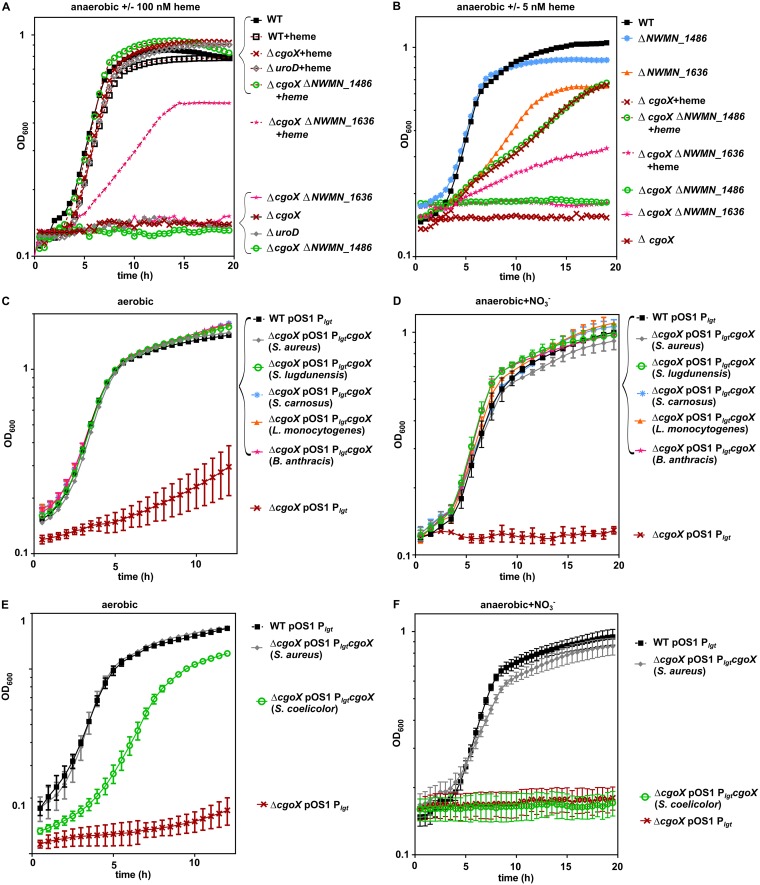
Anaerobic production of heme relies only on CgoX, which is functionally conserved among *Firmicutes*. S. aureus WT and indicated mutants were grown in medium with glycerol as the primary carbon source and nitrate added as indicated. (A) Anaerobic growth with 100 nM exogenous heme added. (B) Anaerobic growth with 5 nM exogenous heme added. (C and D) Aerobic and anaerobic growth of the Δ*cgoX* strain complemented with *cgoX* of various species of *Firmicutes*. (E and F) Aerobic and anaerobic growth of the Δ*cgoX* strain complemented with *cgoX* of the *Actinobacteria* species S. coelicolor.

CgoX appears to be the sole enzyme required for the conversion of coproporphyrinogen to coproporphyrin anaerobically, yet the Δ*NWMN_1636* mutant had a modest growth defect anaerobically. We therefore investigated whether NWMN_1486 or NWMN_1636 makes any contribution to anaerobic heme synthesis using the Δ*cgoX* Δ*NWMN_1636* and Δ*cgoX* Δ*NWMN_1486* mutants. Addition of 100 nM exogenous heme complemented the anaerobic growth of the Δ*cgoX*, Δ*uroD*, and Δ*cgoX* Δ*NWMN_1486* mutants and partly complemented the growth of the Δ*cgoX* Δ*NWMN_1636* mutant (Fig. 2A and Fig. S4A). We next assessed growth of the double mutants using a low concentration of heme to test whether *NWMN_1486* or *NWMN_1636* contributes to heme synthesis in the absence of *cgoX*. We chose 5 nM heme in case the 100 nM exogenous heme was in such excess that it masked any small effects. The growth of the Δ*cgoX* mutant was partly complemented by 5 nM exogenous heme, and the Δ*cgoX* Δ*NWMN_1486* mutant grew indistinguishably when 5 nM heme was provided (Fig. 2B and Fig. S4B). These data suggest that NWMN_1486 has no role in anaerobic heme synthesis. Based on sequence homology, NWMN_1486 may be a heme chaperone (21) and does not appear to contribute to heme synthesis, but the contribution of this protein to S. aureus physiology remains to be explored. The Δ*cgoX* Δ*NWMN_1636* mutant was partly complemented by 5 nM heme but still had a reduced growth rate compared to that of Δ*cgoX* mutant supplemented with heme. These data are consistent with the Δ*NWMN_1636* mutant having a general growth defect unrelated to heme synthesis. The growth defect of the Δ*NWMN_1636* mutant was complemented by providing *NWMN_1636* in *trans*, while exogenous heme had only a modest effect on its growth (Fig. S5). It is possible that NWMN_1636 has some basal coproporphyrinogen III oxidase activity or somehow otherwise contributes to heme synthesis, as heme does increase the growth yield of the Δ*NWMN_1636* mutant. However, the absolute inability of the Δ*cgoX* mutant to grow aerobically or anaerobically (Fig. 1) suggests that any contribution of NWMN_1636 to heme synthesis is minimal. Thus, the function of the DUF1444 family member NWMN_1636 is still unknown.

10.1128/mSphere.00235-19.4 FIG S4 Growth curve of Fig. 2A and B shown with standard errors of the means. Download FIG S4, TIF file, 1.1 MB. Copyright © 2019 Choby and Skaar. 2019 Choby and Skaar This content is distributed under the terms of the Creative Commons Attribution 4.0 International license.

10.1128/mSphere.00235-19.5 FIG S5 The growth defect of the Δ*NWMN_1636* mutant is complemented by expression of gene in *trans* but not completely by heme. Download FIG S5, TIF file, 0.3 MB. Copyright © 2019 Choby and Skaar. 2019 Choby and Skaar This content is distributed under the terms of the Creative Commons Attribution 4.0 International license.

Having established that CgoX functions in both aerobic and anaerobic heme-dependent growth, we tested whether the anaerobic function of CgoX was widespread among organisms that rely on coproporphyrin-dependent heme synthesis. The *cgoX* gene of the related *Firmicutes* species Staphylococcus lugdunensis, Staphylococcus carnosus, Listeria monocytogenes, and Bacillus anthracis complemented the aerobic and anaerobic growth defect of the S. aureus Δ*cgoX* mutant when provided in *trans* (Fig. 2C and D), suggesting that many members of the *Firmicutes* phylum use CgoX for aerobic and anaerobic heme synthesis. Many species of the phylum *Actinobacteria* also synthesize heme via the coproporphyrin intermediate. Interestingly, the *cgoX* gene of Streptomyces coelicolor was able to partly complement the aerobic growth of the S. aureus Δ*cgoX* mutant, suggesting that the enzyme is expressed but was unable to complement growth anaerobically (Fig. 2E and F). These data suggest that the anaerobic function of CgoX is not conserved across all organisms that synthesize heme via coproporphyrin. The inability of S. coelicolor *cgoX* to complement anaerobically is consistent with differences noted in the terminal steps of *Actinobacteria* heme synthesis. For instance, CpfC from *Firmicutes* does not require a cofactor while CpfC of *Actinobacteria* possess an iron-sulfur cluster, and ChdC in *Firmicutes* has a small lip near the active site absent in *Actinobacteria* (10). In conclusion, we have experimentally shown that CgoX is responsible for both aerobic and anaerobic heme synthesis in S. aureus, as well as in many heme-synthesizing species of the *Firmicutes* phylum. More work is needed to identify the electron acceptor CgoX uses anaerobically and to understand more fully the terminal steps of *Actinobacteria* heme synthesis.

### Methods. (i) General growth and reagents.

For bacterial strains, plasmids, and primers, see the supplemental material (Tables S1 to S3, respectively, and references 25 and 26). S. aureus strains were grown routinely on tryptic soy agar (TSA) or broth (TSB) supplemented with 10 μg/ml chloramphenicol when necessary. When used, hemin chloride (referred to as heme) was used at concentrations noted in the text or on figures. Heme was prepared fresh at 10 mM in 0.1 M NaOH; for experiments in which heme was used, an equal volume of 0.1 M NaOH was used for all conditions. Escherichia coli strains were grown on lysogeny broth (LB) or LB agar (LBA), supplemented with 50 μg/ml carbenicillin when necessary. For growth in aerobic liquid medium, an Innova44 incubator with shaking at 180 rpm was used. For standard cultures of 2 to 3 ml, 15-ml round-bottomed polypropylene tubes with aeration lids were used, at a 45° angle in the aerobic incubator or upright and without shaking in the anaerobic incubator. Unless noted otherwise, all chemicals are from Sigma. All molecular biology reagents were from New England Biolabs and used according to the manufacturer’s instructions, unless otherwise noted. Phusion 2X high-fidelity master mix was used for all PCRs for cloning.

10.1128/mSphere.00235-19.6 TABLE S1 Strains used in this study. Download Table S1, DOCX file, 0.01 MB. Copyright © 2019 Choby and Skaar. 2019 Choby and Skaar This content is distributed under the terms of the Creative Commons Attribution 4.0 International license.

10.1128/mSphere.00235-19.7 TABLE S2 Plasmids used in this study. Download Table S2, DOCX file, 0.01 MB. Copyright © 2019 Choby and Skaar. 2019 Choby and Skaar This content is distributed under the terms of the Creative Commons Attribution 4.0 International license.

10.1128/mSphere.00235-19.8 TABLE S3 Primers used in this study. Download Table S3, DOCX file, 0.01 MB. Copyright © 2019 Choby and Skaar. 2019 Choby and Skaar This content is distributed under the terms of the Creative Commons Attribution 4.0 International license.

**(ii) Gene deletions.** In-frame, unmarked deletions were created by allelic exchange as described in Bae and Schneewind (23), with some modifications. The pKOR1 plasmids containing ∼1-kb homologous regions flanking upstream and downstream of the gene to be deleted were prepared using NEB HiFi assembly according to manufacturer’s suggestions. The pKOR1 backbone was amplified by PCR using primers JC291/JC292, which produce a linear product not including the *attB* recombination sites. The ∼1-kb flanking regions were amplified from S. aureus Newman genomic DNA. During allelic exchange, 2 μM heme was added to the medium after generation of merodiploids to chemically complement heme synthesis defects. Deletions were confirmed by PCR using isolated genomic DNA, and phenotypes were complemented by providing the gene in *trans.* For *uroD,* flanking regions were amplified using primers JC419/JC420 (upstream flanking) and JC421/JC422 (downstream flanking). For *cgoX*, flanking regions were amplified using primers JC631/JC632 (upstream flanking) and JC633/JC508 (downstream flanking). Deletions of *uroD* and *cgoX* were confirmed using primers JC427/JC428. For *NWMN_1636*, flanking regions were amplified using JC621/JC622 (upstream flanking) and JC623/JC624 (downstream flanking). The deletion was confirmed with PCR using primers JC627/JC628. For *NWMN_1486*, flanking regions were amplified using JC617/JC618 (upstream flanking) and JC619/JC620 (downstream flanking). The deletion was confirmed with PCR using primers JC625/JC626. To create Δ*cgoX* Δ*NWMN_1636,* pKOR1-*cgoX* was transduced as described previously (24) using bacteriophage ϕ85 into Δ*NWMN_1636*, and allelic exchange was performed. To create Δ*cgoX* Δ*NWMN_1486,* pKOR1-*NWMN_1486* was transduced into Δ*cgoX*, and allelic exchange was performed, with 2 μM heme supplemented for all steps.

**(iii) Genetic complementation.** To complement the Δ*NWMN_1636* mutant phenotypes, *NWMN_1636* was cloned from S. aureus Newman genomic DNA using primers JC644/JC645 with homology to pOS1 P*_lgt_* digested with NdeI and BamHI (NEB) and ligated using NEB HiFi assembly mix. To complement the Δ*cgoX* mutant phenotypes, *cgoX* was cloned from S. aureus Newman genomic DNA using primers JC515/JC516 to incorporate a C-terminal FLAG tag with homology to pOS1 P*_lgt_* digested with XhoI and BamHI (NEB) and ligated using NEB HiFi assembly mix. The *cgoX* genes of other species were cloned in the same manner, except that nested PCR was used to accommodate the length of the 3′ primer incorporating the FLAG tag sequence, as noted below. Genomic DNA was used as the template for *Firmicutes* species, while pET15b-*cgoX* (S. coelicolor) (provided by the Dailey lab) was used as the template for S. coelicolor *cgoX*. For S. carnosus, primers JC735/JC736 were used to amplify *cgoX* from the genome and became the template for JC735/JC737 to amplify the final product; for S. lugdunensis, primers JC738/7JC39 were used to amplify *cgoX* from the genome and became the template for JC738/JC740 to amplify the final product; for L. monocytogenes, primers JC744/JC745 were used to amplify *cgoX* from the genome and became the template for JC744/JC746 to amplify the final product; for B. anthracis, primers JC747/JC748 were used to amplify *cgoX* from the genome and became the template for JC747/JC749 to amplify the final product. S. coelicolor *cgoX* did not require a nested PCR and was amplified with primers JC791/JC792. This plasmid was sequenced to confirm its accuracy. The complementation plasmids were confirmed by restriction digest after isolation from DH5α following transformation, transformed into S. aureus RN4220 by electroporation, and transduced as described previously (24) using bacteriophage ϕ85.

**(iv) Growth curves.** Growth curves were performed using overnight cultures of biological triplicates or quadruplicates depending on the experiment. After overnight growth, 1 μl of overnight culture was added to 199 μl of RPMI medium (no glucose; Gibco) supplemented with 1% Casamino Acids, 0.04% glycerol, and 40 mM nitrate (from sodium nitrate; Fisher) in a 96-well round-bottomed plate (Costar), and growth was monitored in a BioTek plate reader with shaking at 37°C. Data were graphed using GraphPad Prism. Three independent experiments were performed (with the exception of data showing complementation with 100 nM heme, which was from a single representative experiment), the mean of the biological triplicates or quadruplicates was calculated, and the three means were graphed with the standard errors of the means shown for each figure panel, except for panels in which no error bars are displayed for ease of viewing. The same data, with errors indicated, are presented as supplemental figures. For aerobic growth curves, strains were streaked to TSA and grown aerobically for 24 h at 37°C. Single colonies were used (except for heme-deficient strains, for which a few colonies were used) to inoculate overnight cultures in 3 ml of TSB in aeration tubes and grown for 15 h with shaking at 180 rpm in an Innova44 incubator at 37°C. For anaerobic experiments, a Coy anaerobic chamber was used, filled with a mix of 90% nitrogen, 5% carbon dioxide, and 5% hydrogen gases, and hydrogen levels were monitored to ensure a minimum of 2% hydrogen concentration. Palladium catalysts (Coy) were used to remove any residual oxygen by reaction with hydrogen. A Coy static incubator was maintained at 37°C. Solutions and plasticware were allowed to equilibrate for >24 h inside the glove box before use. For anaerobic samples, strains were streaked to TSA and grown aerobically for 24 h at 37°C. TSA plates were moved into the anaerobic chamber, and single colonies were used (except for heme-deficient strains, for which a few colonies were used) to inoculate overnight cultures in 2 ml of anaerobic TSB in aeration tubes and grown for 16 h anaerobically without shaking at 37°.
