# Supplementary figures and images for: Staphylococcus aureus Coproporphyrinogen III Oxidase Is Required for Aerobic and Anaerobic Heme Synthesis
Source: mSphere. 2019 Jul 10;4(4):e00235-19. doi: 10.1128/mSphere.00235-19 (PMC6620371; doi:10.1128/mSphere.00235-19)

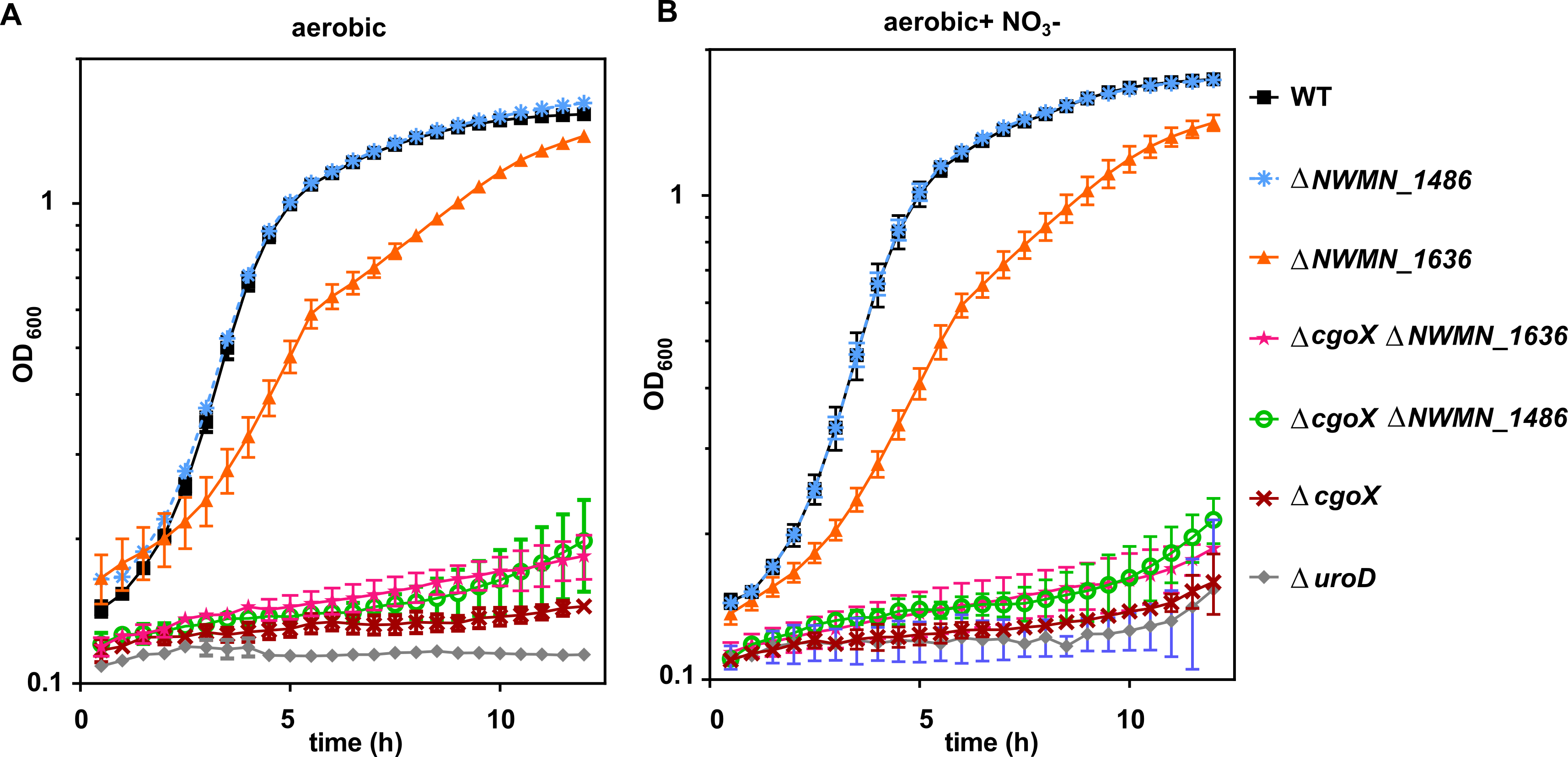

Supplement: FIG S1 [file mSphere.00235-19-sf001.tif]

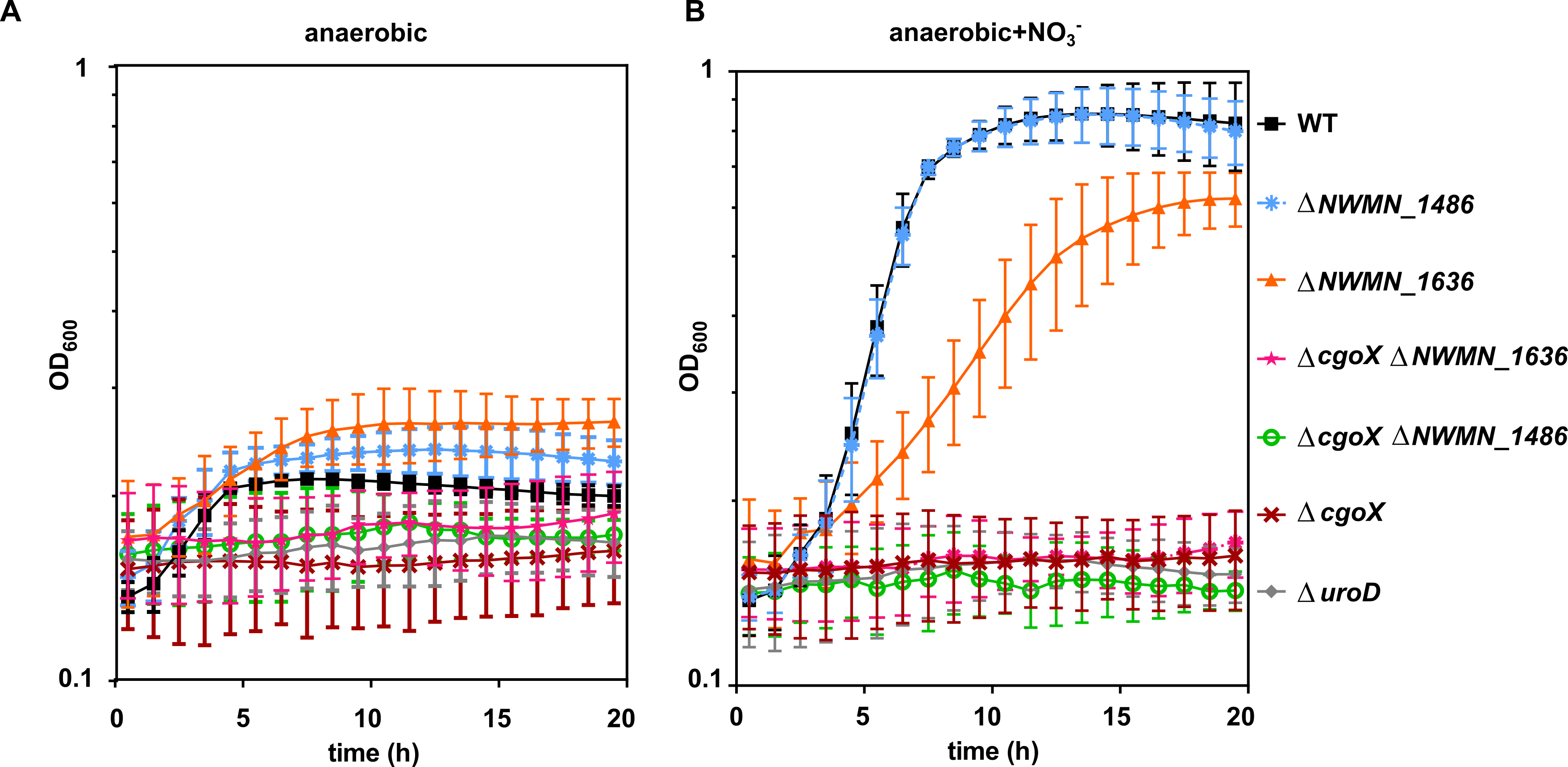

Supplement: FIG S2 [file mSphere.00235-19-sf002.tif]

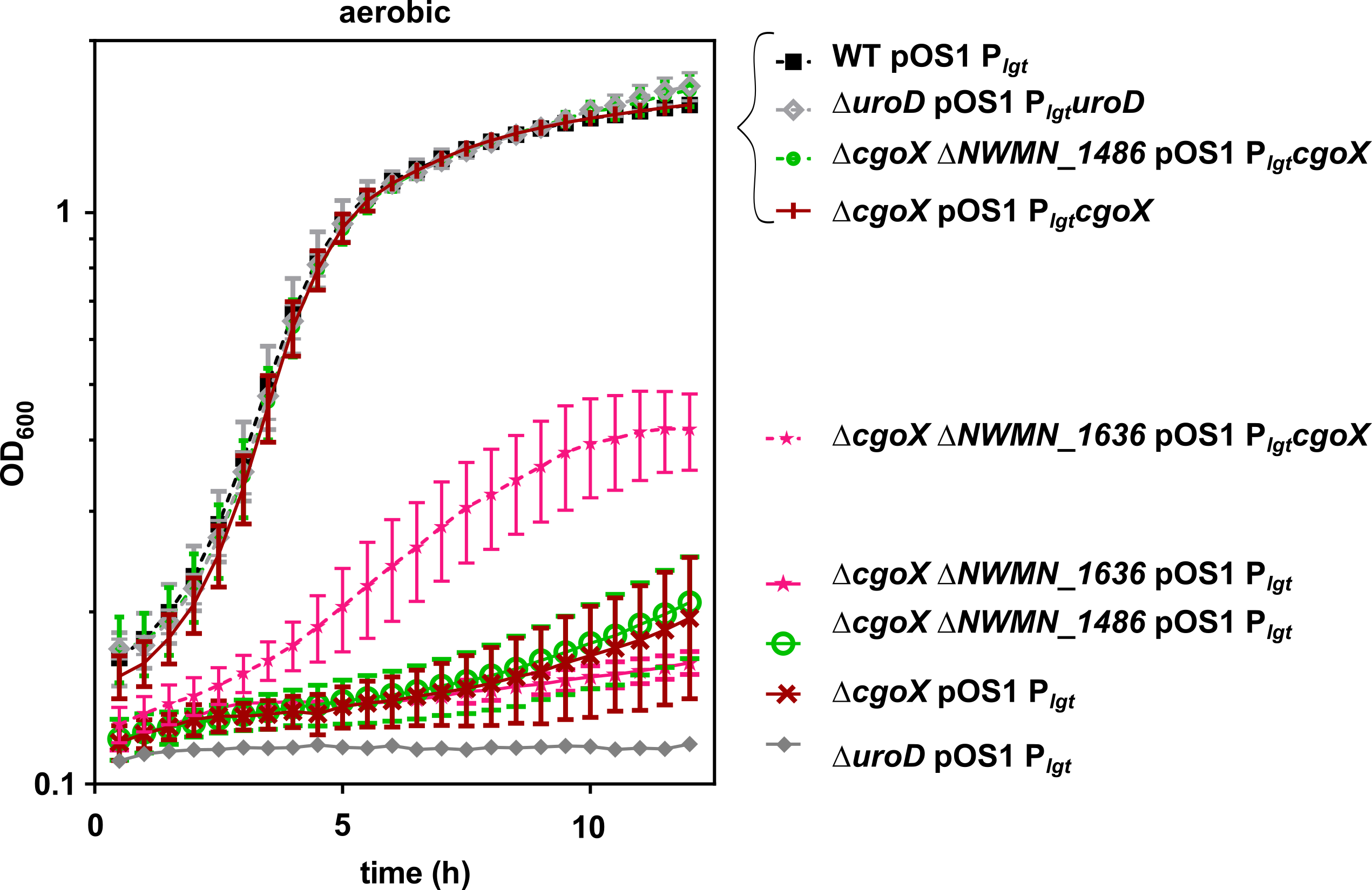

Supplement: FIG S3 [file mSphere.00235-19-sf003.tif]

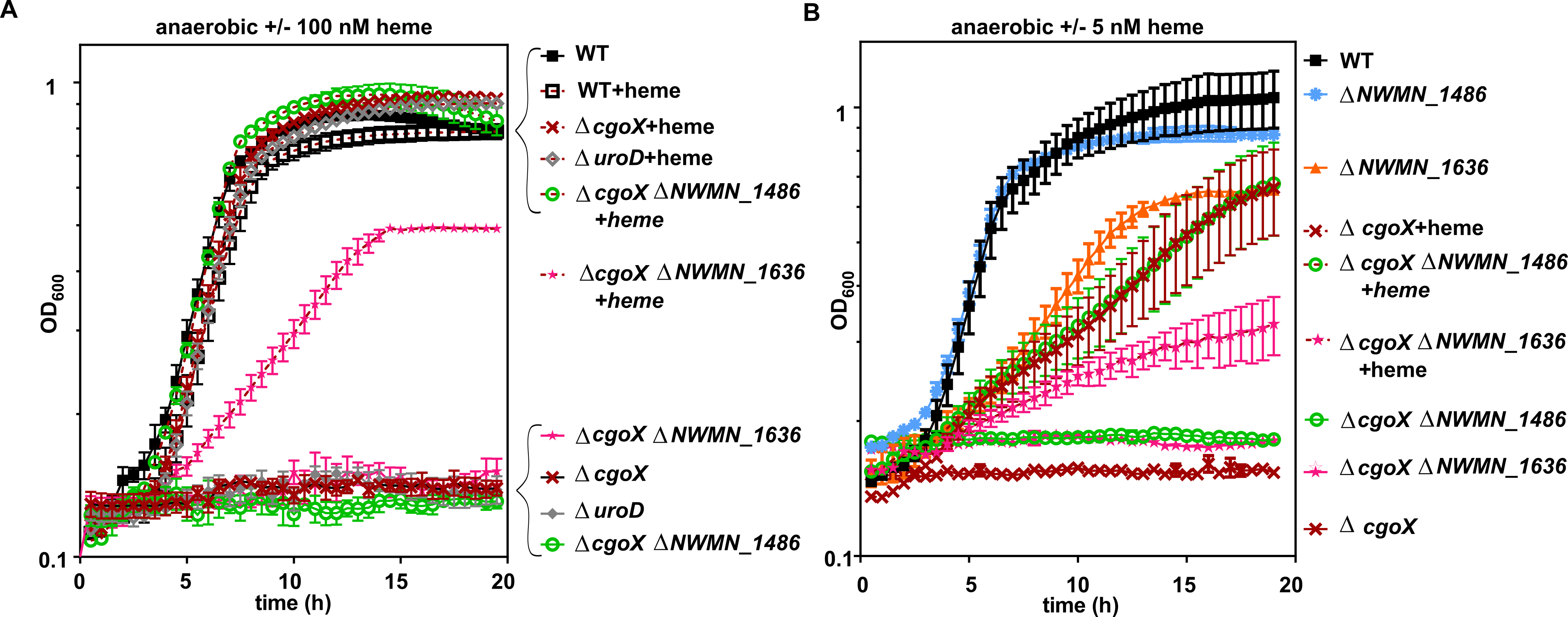

Supplement: FIG S4 [file mSphere.00235-19-sf004.tif]

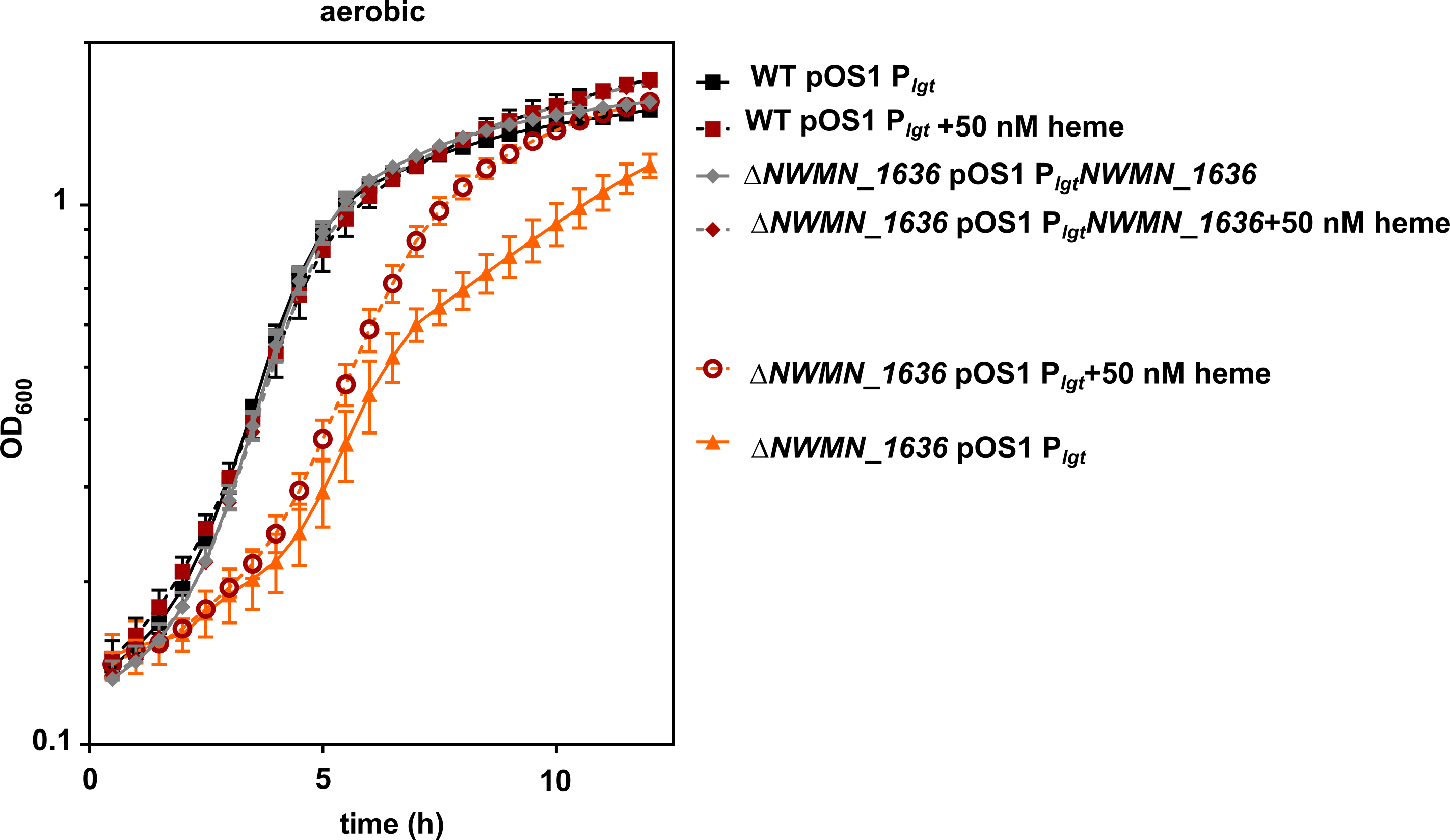

Supplement: FIG S5 [file mSphere.00235-19-sf005.tif]
