# Supplementary material for: Staphylococcus aureus Coproporphyrinogen III Oxidase Is Required for Aerobic and Anaerobic Heme Synthesis
Source: mSphere. 2019 Jul 10;4(4):e00235-19. doi: 10.1128/mSphere.00235-19 (PMC6620371; doi:10.1128/mSphere.00235-19)
Supplement: TABLE S1 [file mSphere.00235-19-st001.docx]

| **Table S1 Strains** | | | |
| --- | --- | --- | --- |
| **Species** | **Genotype** | **Description** | **Reference** |
| *S. aureus* strain Newman | WT | Wildtype laboratory stock | ([25](#_ENREF_25)) |
| *S. aureus* strain Newman | Δ*uroD* | In frame deletion of *NWMN_1725* | This study |
| *S. aureus* strain Newman | Δ*cgoX* | In frame deletion of *NWMN_1723* | This study |
| *S. aureus* strain Newman | Δ*NWMN_1486* | In frame deletion of *NWMN_1486* | This study |
| *S. aureus* strain Newman | Δ*NWMN_1636* | In frame deletion of *NWMN_1636* | This study |
| *S. aureus* strain Newman | Δ*cgoX* Δ*NWMN_1486* | In frame deletion of *NWMN_1723* and *NWMN_1486* | This study |
| *S. aureus* strain Newman | Δ*cgoX* Δ*NWMN_1636* | In frame deletion of *NWMN_1723* and *NWMN_1636* | This study |
