# Supplementary material for: Staphylococcus aureus Coproporphyrinogen III Oxidase Is Required for Aerobic and Anaerobic Heme Synthesis
Source: mSphere. 2019 Jul 10;4(4):e00235-19. doi: 10.1128/mSphere.00235-19 (PMC6620371; doi:10.1128/mSphere.00235-19)
Supplement: TABLE S2 [file mSphere.00235-19-st002.docx]

| **Table S2 Plasmids** | | |
| --- | --- | --- |
| **Plasmid** | **Description** | **Reference** |
| pKOR1 | Allelic exchange vector for *S. aureus* | ([23](#_ENREF_23)) |
| pKOR1-*uroD* | Vector to delete *uroD* | This study |
| pKOR1-*cgoX* | Vector to delete *cgoX* | This study |
| pKOR1-*NWMN_1636* | Vector to delete *NWMN_1636* | This study |
| pKOR1-*NWMN_1486* | Vector to delete *NWMN_1486* | This study |
| pOS1 P*_lgt_* | Complementation vector with constitutive promoter | ([26](#_ENREF_26)) |
| pOS1 P*_lgt_uroD* | *uroD* complementation vector | This study |
| pOS1 P*_lgt_cgoX* | *S. aureus cgoX*_*FLAG* complementation vector | This study |
| pOS1 P*_lgt_cgoX (S. carnosus)* | *S. carnosus cgoX*_*FLAG* complementation vector; strain TM300, *SCA_1406* | This study |
| pOS1 P*_lgt_cgoX (S. lugdunensis)* | *S. lugdunensis cgoX*_*FLAG* complementation vector; strain Hong Kong, *SLGD_01131* | This study |
| pOS1 P*_lgt_cgoX (L. monocytogenes)* | *L. monocytogenes cgoX*_*FLAG* complementation vector; strain EDG-e, *lmo0884* | This study |
| pOS1 P*_lgt_cgoX (B. anthracis)* | *B. anthracis cgoX*_*FLAG* complementation vector; strain Sterne, *BAS1001* | This study |
| pOS1 P*_lgt_cgoX (S. coelicolor)* | *S. coelicolor cgoX*_*FLAG* complementation vector; corresponds to *SCO6041* | This study |
