# Supplementary material for: Staphylococcus aureus Coproporphyrinogen III Oxidase Is Required for Aerobic and Anaerobic Heme Synthesis
Source: mSphere. 2019 Jul 10;4(4):e00235-19. doi: 10.1128/mSphere.00235-19 (PMC6620371; doi:10.1128/mSphere.00235-19)
Supplement: TABLE S3 [file mSphere.00235-19-st003.docx]

| **Table S3 Primers** | |
| --- | --- |
| **Primer name** | **Sequence** |
| JC291 | GGGCCCGAGCTTAAGACT |
| JC292 | GATATCCCCTATAGTGAGTCGTATTAC |
| JC419 | TATAGGGGATATCTGACATTTATTATAAAACATATGACG |
| JC420 | TAAAACTATGATATGCACCATAAAGGCCTC |
| JC421 | TATGGTGCATATCATAGTTTTACATTGAAGCAC |
| JC422 | GGCCAGTCTTAAGCTCGGGCCCAACGTTTCACGCTTCTTTC |
| JC631 | CGACTCACTATAGGGGATATCTTATTAGCACCATGGAATG |
| JC632 | CATCCAACAATTAACAACGTTTCACGCTTCTTTC |
| JC633 | GAAGCGTGAAACGTTGTTAATTGTTGGATGGTGTTG |
| JC508 | GACGGCCAGTCTTAAGCTCGGGCCCAACGCCTATACCTTCTGAAAAAG |
| JC427 | GCAATGGTATCAATCGGGACTTAAAAC |
| JC428 | GAATGGTTCAGGACAGAGTCGAA |
| JC621 | CGACTCACTATAGGGGATATCGAGATACAATTGTACCTCACAC |
| JC622 | CTTATCCAATTATTTATGTAACACTCCTAATTTCGC |
| JC623 | TTAGGAGTGTTACATAAATAATTGGATAAGGAGTTTTG |
| JC624 | AACGACGGCCAGTCTTAAGCTCGGGCCCTAATGTGAATGATCTTTATATTTTATACTTT |
| JC627 | GGGCACCAGGTTTTGAAGAAGAAG |
| JC628 | CTGAAGGTTTACTTACAGGGATACGACC |
| JC617 | CGACTCACTATAGGGGATATCTTACCTTTAGCTGAAGTTGTATTTG |
| JC618 | AAATTTCAATTTTTTTTTAATGCCCCGCTTCTC |
| JC619 | AAGCGGGGCATTAAAAAAAAATTGAAATTTCGAGTCTTTAAC |
| JC620 | AAAACGACGGCCAGTCTTAAGCTCGGGCCCTCACAGCGATTTGACCTTTTAAC |
| JC625 | GCGAAAATATCATTTCTGTCCCACTCCC |
| JC626 | GTGCACGTTCTATATTGTCTATTGCTGG |
| JC644 | AAATACAATTGAGGTGAACATATGAATACCTTTCAAATGAGAGATAAATTAAAG |
| JC645 | AAACACTACCCCCTTGTTTGGATCCCTATTTATCTTTATTAAATTTACGACG |
| JC515 | GCGCCTCGAGCGCGTGACTAAATCAGTGGCTATTATAG |
| JC516 | GCGCGGATCCTTACTTATCGTCATCATCTTTATAGTCCAACTCTGCGATTACTTCTTC |
| JC735 | CAATTGAGGTGAACATATGCTCGAGATGACTAAAAAAATTGCAATTATC |
| JC736 | GATCCTTACTTATCGTCATCATCTTTATAGTCTTTCACGGTAGCAGCTAATTC |
| JC737 | AAACACTACCCCCTTGTTTGGATCCTTACTTATCGTCATCATCTTTATAGTC |
| JC738 | CAATTGAGGTGAACATATGCTCGAGGTGAGCAAAAAAATTGCG |
| JC739 | TTGGATCCTTACTTATCGTCATCATCTTTATAGTCTAATTCATGATATATTTCATCAGC |
| JC740 | AAACACTACCCCCTTGTTTGGATCCTTACTTATCGTCATCATCTTTATAGTC |
| JC744 | CAATTGAGGTGAACATATGCTCGAGGTGAAACATATAGTCATTATCGG |
| JC745 | GATCCTTACTTATCGTCATCATCTTTATAGTCCACCTCTTCCAAGTAATCTACTAATTC |
| JC746 | AAACACTACCCCCTTGTTTGGATCCTTACTTATCGTCATCATCTTTATAGTC |
| JC747 | CAATTGAGGTGAACATATGCTCGAGTTGAGGAAAAAAGTTGTAATCATC |
| JC748 | GATCCTTACTTATCGTCATCATCTTTATAGTCTTGTGCGATTAATTCCGTATTC |
| JC749 | AAACACTACCCCCTTGTTTGGATCCTTACTTATCGTCATCATCTTTATAGTC |
| JC791 | AAATACAATTGAGGTGAACATATGCTCGAGATGAGCGCAAGTGAGACCCCG |
| JC792 | AAAGGGGGAAACACTACCCCCTTGTTTGGATCCTTATTCTCCCGCTCCGCCGTG |
